# Supplementary material for: Authentication and Geographical Characterisation of Italian Grape Musts through Glucose and Fructose Carbon Isotopic Ratios Determined by LC-IRMS
Source: Molecules. 2023 Feb 2;28(3):1411. doi: 10.3390/molecules28031411 (PMC9919784; doi:10.3390/molecules28031411)
Supplement: Supplementary file 1 [file molecules-28-01411-s001.zip › molecules-2144398-supplementary.pdf]

Table S1: Origin Area, region of provenance, variety, sampling date and $\delta^{13}C_{GLUCOSE}$, $\delta^{13}C_{FRUCTOSE}$ and $R_{13C\_G/F}$ of the authentic Italian must samples

**Origin Area**	**Italian Region**	**Variety**	**Sampling date**	**δ^{13}C (‰, vs V-PDB) glucose**	**δ^{13}C (‰, vs V-PDB) fructose**	**$R_{13C\_G/F}$**
North	Trentino-Alto Adige	Chardonnay	31/08/2021	-29.2	-27.8	1.1
North	Trentino-Alto Adige	Pinot	14/09/2021	-27.7	-27.1	1.0
North	Trentino-Alto Adige	Teroldego	21/09/2021	-26.3	-27.3	1.0
North	Trentino-Alto Adige	Nosiola	21/09/2021	-27.5	-26.9	1.0
North	Trentino-Alto Adige	Nosiola	21/09/2021	-27.9	-27.1	1.0
North	Friuli-Venezia Giulia	Verduzzo	09/11/2021	-28.8	-29.6	1.0
North	Friuli-Venezia Giulia	Sauvignon	10/09/2021	-27.2	-25.7	1.1
North	Friuli-Venezia Giulia	Malvasia	16/09/2021	-25.5	-24.0	1.1
North	Friuli-Venezia Giulia	Chardonnay	07/09/2021	-26.0	-24.9	1.0
North	Friuli-Venezia Giulia	Chardonnay	07/09/2021	-28.3	-27.0	1.0
North	Friuli-Venezia Giulia	Sauvignon	07/09/2021	-29.1	-27.6	1.1
North	Friuli-Venezia Giulia	Chardonnay	07/09/2021	-28.4	-26.4	1.1
North	Friuli-Venezia Giulia	Pinot	07/09/2021	-27.9	-28.8	1.0
North	Veneto	Garganega	20/09/2021	-26.4	-25.5	1.0
North	Veneto	Pinot	09/09/2021	-28.8	-27.7	1.0
North	Veneto	Glera	20/09/2021	-29.4	-28.6	1.0
North	Veneto	Glera	21/09/2021	-25.9	-25.4	1.0
North	Veneto	Merlot	15/09/2021	-28.3	-27.4	1.0
North	Veneto	Merlot	21/09/2021	-27.8	-25.7	1.1
North	Veneto	Pinot	06/09/2021	-27.0	-27.6	1.0
North	Piedmont	Chardonnay	04/09/2021	-25.8	-26.5	1.0
North	Piedmont	Moscato	17/09/2021	-28.8	-26.9	1.1
North	Piedmont	Erbaluce	22/09/2021	-29.8	-27.8	1.1
North	Piedmont	Moscato	02/09/2021	-24.8	-25.0	1.0
North	Piedmont	Barbera	23/09/2021	-26.1	-25.6	1.0

North	Piedmont	Nebbiolo	11/10/2021	-26.5	-26.6	1.0
North	Piedmont	Dolcetto	13/09/2021	-27.6	-26.6	1.0
North	Lombardy	Chardonnay	24/08/2021	-26.9	-27.1	1.0
North	Lombardy	Lambrusco	04/10/2021	-29.5	-27.0	1.1
North	Lombardy	Cabernet	01/10/2021	-28.0	-27.7	1.0
North	Lombardy	Nebbiolo	10/10/2021	-27.0	-26.2	1.0
North	Lombardy	Nebbiolo	10/10/2021	-26.6	-26.3	1.0
North	Lombardy	Trebbiano	22/09/2021	-27.8	-25.9	1.1
North	Lombardy	Barbera	17/09/2021	-28.1	-27.6	1.0
North	Lombardy	Pinot	26/09/2021	-27.9	-27.3	1.0
Centre	Emilia-Romagna	Lambrusco	21/09/2021	-28.2	-28.2	1.0
Centre	Emilia-Romagna	Lambrusco	17/09/2021	-24.4	-23.4	1.0
Centre	Emilia-Romagna	Lambrusco	21/09/2021	-24.0	-21.9	1.1
Centre	Emilia-Romagna	Lambrusco	03/09/2021	-23.9	-22.2	1.1
Centre	Emilia-Romagna	Pignoletto	02/09/2021	-23.6	-22.7	1.0
Centre	Emilia-Romagna	Albana	27/08/2021	-26.8	-27.1	1.0
Centre	Emilia-Romagna	Albana	03/09/2021	-25.5	-25.2	1.0
Centre	Lazio	Sangiovese	09/09/2021	-27.5	-28.5	1.0
Centre	Lazio	Grechetto	22/09/2021	-27.4	-27.8	1.0
Centre	Lazio	Moscato	22/09/2021	-22.3	-22.7	1.0
Centre	Lazio	Trebbiano	17/09/2021	-24.6	-24.5	1.0
Centre	Lazio	Sauvignon	26/09/2021	-27.4	-26.9	1.0
Centre	Tuscany	Sangiovese	22/09/2021	-22.7	-23.0	1.0
Centre	Tuscany	Sangiovese	28/09/2021	-25.1	-23.2	1.1
Centre	Tuscany	Sangiovese	21/09/2021	-28.0	-27.0	1.0
Centre	Tuscany	Ansonica	14/10/2021	-24.9	-25.4	1.0
Centre	Tuscany	Cabernet	08/09/2021	-23.6	-23.2	1.0
Centre	Tuscany	Cabernet	08/09/2021	-23.8	-24.0	1.0
Centre	Abruzzo	Montepulciano	21/10/2021	-23.4	-23.6	1.0
Centre	Abruzzo	Passerina	03/09/2021	-24.4	-24.5	1.0
Centre	Abruzzo	Passerina	14/09/2021	-24.8	-24.8	1.0
Centre	Abruzzo	Pinot	23/08/2021	-24.7	-25.0	1.0

Centre	Abruzzo	Sauvignon	20/08/2021	-26.0	-26.4	1.0
Centre	Marche	Verdicchio	05/10/2021	-24.2	-25.0	1.0
Centre	Marche	Lacrima	24/09/2021	-25.0	-23.9	1.0
Centre	Marche	Biancame	01/10/2021	-22.9	-22.6	1.0
Centre	Marche	Chardonnay	23/08/2021	-24.9	-24.7	1.0
Centre	Marche	Verdicchio	20/09/2021	-26.6	-25.2	1.1
Centre	Marche	Verdicchio	23/09/2021	-26.3	-26.2	1.0
Centre	Marche	Pecorino	16/09/2021	-25.7	-25.5	1.0
Centre	Umbria	Trebbiano	16/09/2021	-23.1	-23.2	1.0
Centre	Umbria	Grechetto	17/09/2021	-27.3	-27.4	1.0
Centre	Umbria	Trebbiano	20/09/2021	-24.8	-26.3	0.9
Centre	Umbria	Cabernet	16/09/2021	-22.8	-22.0	1.0
South	Campania	Fiano	13/10/2021	-25.1	-27.2	0.9
South	Campania	Caprettone	12/10/2021	-28.7	-27.7	1.0
South	Campania	Fiano	30/09/2021	-25.4	-24.8	1.0
South	Campania	Aglianico	14/10/2021	-27.3	-25.6	1.1
South	Campania	Lambrusco	30/09/2021	-25.2	-24.7	1.0
South	Basilicata	Pinot	13/10/2021	-27.1	-26.8	1.0
South	Basilicata	Cabernet	15/10/2021	-27.8	-27.5	1.0
South	Basilicata	Sangiovese	13/10/2021	-28.0	-27.3	1.0
South	Basilicata	Malvasia	06/10/2021	-26.1	-26.1	1.0
South	Basilicata	Primitivo	26/08/2021	-25.7	-24.8	1.0
South	Apulia	Negro	27/09/2021	-23.7	-23.0	1.0
South	Apulia	Chardonnay	26/08/2021	-26.1	-24.9	1.0
South	Apulia	Chardonnay	17/08/2021	-26.0	-25.7	1.0
South	Apulia	Chardonnay	26/08/2021	-28.9	-27.7	1.0
South	Apulia	Aleatico	11/09/2021	-24.6	-24.4	1.0
South	Sardinia	Monica	12/09/2021	-25.9	-25.1	1.0
South	Sardinia	Cannonau	31/08/2021	-24.8	-24.9	1.0
South	Sardinia	Malvasia	31/08/2021	-25.4	-24.4	1.0
South	Sardinia	Torbato	02/09/2021	-25.8	-25.2	1.0
South	Sardinia	Vermentino	02/09/2021	-25.0	-25.5	1.0

South	Sicily	Carricante	16/09/2021	-24.2	-24.4	1.0
South	Sicily	Viogner	10/09/2021	-23.1	-21.9	1.1
South	Sicily	Merlot	10/09/2021	-22.8	-23.2	1.0
South	Sicily	Nero	10/10/2021	-26.5	-27.4	1.0
South	Sicily	Catarratto	30/07/2021	-25.1	-25.0	1.0
